# Effective Use of a Combined Video Laryngoscope and Bronchoscope System in the Emergency Department for a Patient With Severe Upper Airway Obstruction Due to Angioedema: A Case Report

**DOI:** 10.7759/cureus.76285

**Published:** 2024-12-23

**Authors:** Saki Miyajima, Kan Takahashi, Sho Matsuba

**Affiliations:** 1 Department of Anesthesiology, Kanazawa Medical University, Ishikawa, JPN

**Keywords:** angioedema, combined video laryngoscope and bronchoscope system, difficult airway management, intubation outside the operating room, quincke's edema

## Abstract

Management of difficult airways in the emergency department is challenging. Herein, we report a case of successful management of severe upper airway obstruction caused by angioedema, where intubation was achieved using a dual-function video laryngoscope and bronchoscope system in the emergency department for a patient with severe upper airway stenosis due to angioedema. A 74-year-old obese man with dyspnea presented to our emergency department. Despite initial attempts using a conventional intubation technique, the patient's airway remained difficult to manage because of marked enlargement of the tongue. The anesthesiologists decided to apply the dual-function video laryngoscope and bronchoscope system (GlideScope Core System™, Verathon Inc., Bothell, WA, USA). Finally, nasotracheal intubation was successfully performed using a bronchoscope switched from a video laryngoscope. The patient's respiratory status improved post-intubation. Subsequent management proceeded uneventfully. The patient was transferred to the ICU, where he was diagnosed with angioedema after admission.

## Introduction

Securing the airway in the emergency department is challenging due to the urgency of the situation, the complexity of unfamiliar cases, and the limited availability of airway equipment, all of which contribute to increased difficulty and a higher rate of complications during intubation [1,2]. Moreover, difficult airway cases are frequently encountered, often with unknown causes. In cases of upper airway edema, the rapid progression of symptoms can lead to asphyxia, necessitating urgent decision-making and the use of appropriate devices to secure the airway. Herein, we report a case of respiratory compromise due to upper airway obstruction caused by Quincke's edema, a type of non-inherited angioedema [3,4], successfully managed using a specialized video laryngoscope and bronchoscope system.

## Case presentation

A 74-year-old male (height 165 cm, weight 104 kg, BMI 38) underwent excision surgery for a sublingual ranula in the oral surgery department. He began experiencing recurrent intra-oral edema after being discharged from the hospital, which was followed at the outpatient for two months. Upon presentation, he exhibited significant tongue and oropharyngeal edema that progressively worsened over several hours, leading to respiratory distress. He was urgently transported to our emergency department. His medical history included hypertension, diabetes mellitus, and hyperlipidemia, for which he was taking telmisartan 40 mg (one tablet/day), amlodipine 5 mg (one tablet/day), and metformin 500 mg (one tablet/day). On arrival, his blood pressure was 147/81 mmHg, heart rate was 75/min, and SpO2 was 98% with a mask that delivered 15 L/min of oxygen. He was exhibiting orthopnea. Emergency physicians made several attempts to intubate the patient using a video laryngoscope; however, insertion of the blade into the oral cavity proved difficult with no bleeding, leading to a request for the anesthesiology team 20 minutes after the patient came to the emergency room. The anesthesiology team rushed to the emergency department with a dual-function bronchoscope and video laryngoscope system (GlideScope Core System™, Verathon Inc., Bothell, WA, USA).

**Figure 1:**
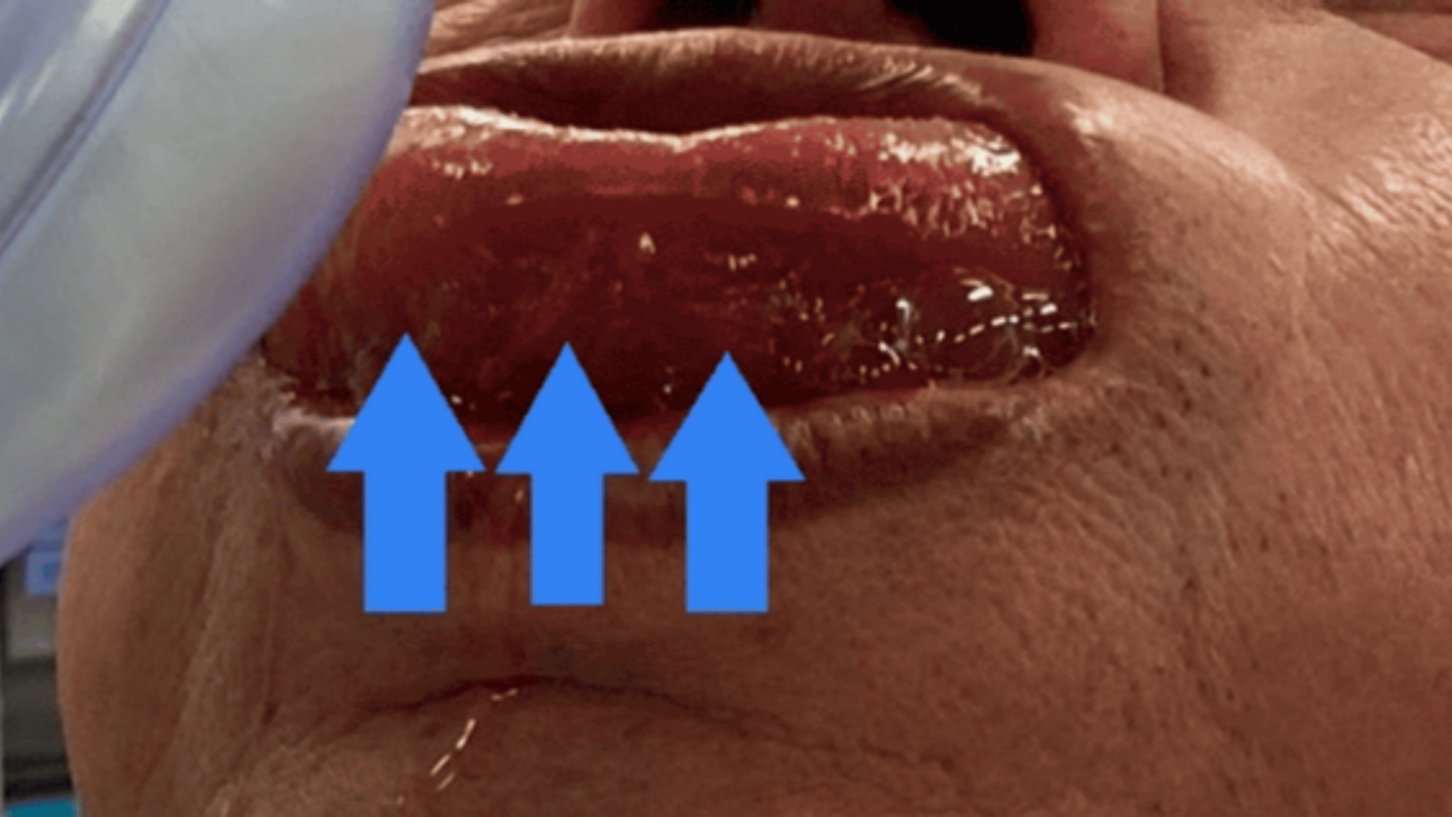
Patient with orthopnea upon the arrival of the anesthesiology team Enlarged tongue protruding from the mouth (arrows)

**Figure 2:**
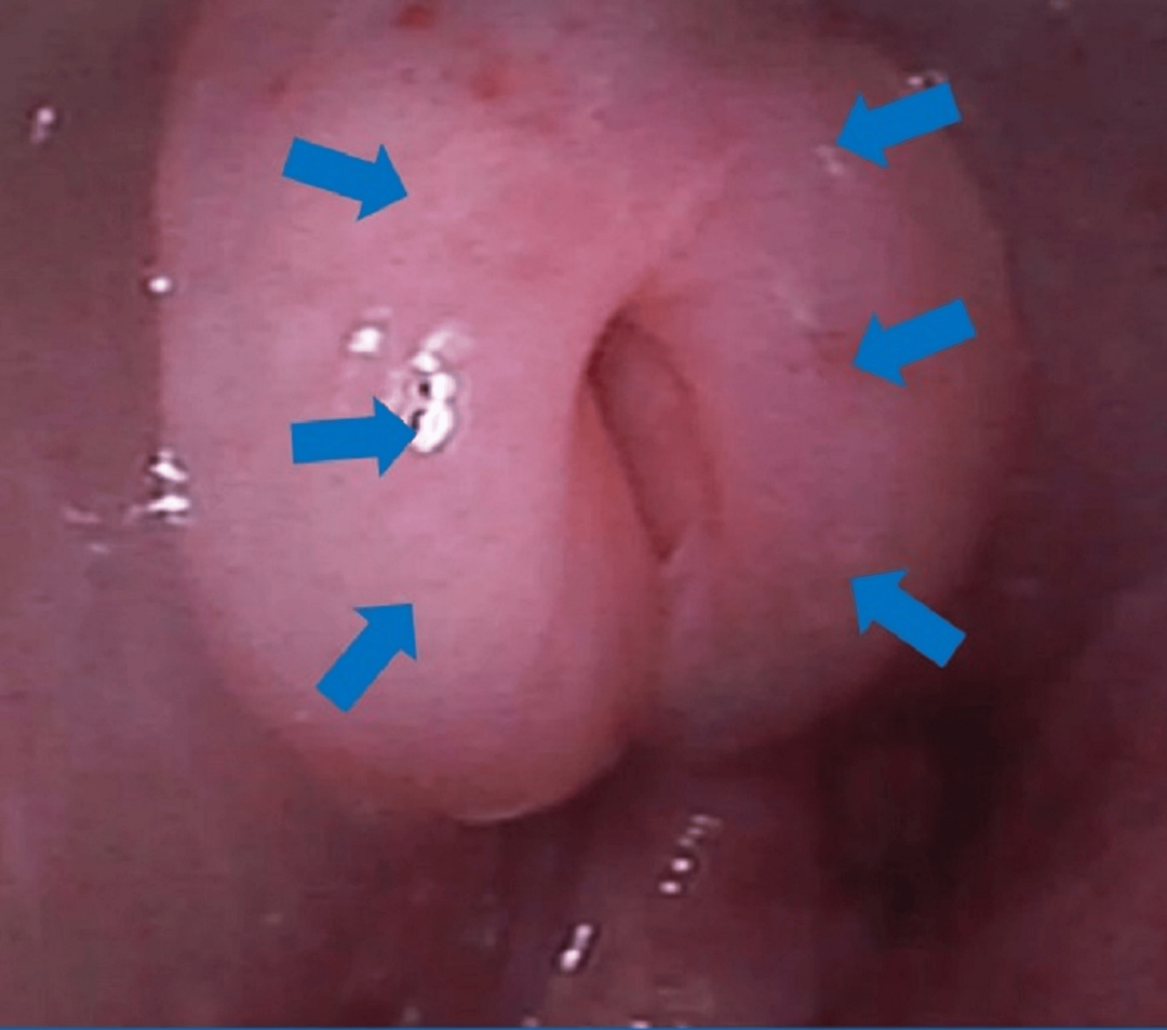
Bronchoscope image showing a severely edematous epiglottis (arrows)

Despite multiple attempts by the senior anesthesiologist, the insertion of the video laryngoscope blade failed due to the patient’s severely edematous and protruded tongue (Figure 1), and the patient's vigorous movement prevented adequate visualization without sedation and paralyzation. While monitoring the patient's spontaneous breathing, we administered 4 mg of midazolam intravenously and advanced a bronchoscope through a wire-reinforced endotracheal tube into the nostril. Notable epiglottic edema was observed under bronchoscopy (Figure 2). After spraying 4% lidocaine around the glottis, nasotracheal intubation was successfully performed. During the procedure, SpO2 was maintained at 96-98% with an oxygen reservoir mask delivering 15 L/min. A spiral tube with a diameter of 7.5 mm was used for intubation, which was fixed at a depth of 29 cm from the left nostril. Following intubation, the patient was transferred to the ICU and underwent open tracheostomy in the operating room under general anesthesia at a later date.

On the first day of hospitalization, an open tracheotomy was performed by otolaryngologists in the OR under general anesthesia. Differential diagnoses included postoperative edema, allergic edema, Quincke's edema, and acquired C1 inhibitor deficiency. As part of the treatment and diagnostic process, telmisartan was discontinued, and antihistamines were administered for 14 days. The patient was administered hydrocortisone at 400 mg/day for the treatment of the pharyngeal and laryngeal edema. Blood tests for C1-inactivator activity were performed to rule out hereditary angioedema, which showed a normal level of 111%. The pharyngeal and laryngeal edema subsequently improved. The patient remained in the ICU for five days, and the tracheotomy was closed after 24 days. He was discharged home after a 28-day hospital stay with almost normal general status.

## Discussion

Angioedema can result from various etiologies [5,6], including allergic reactions, history of oral procedures, medication-induced effects, hereditary factors, and acquired C1 inhibitor deficiency. Angiotensin-converting enzyme inhibitors are a well-known trigger for angioedema, particularly in the absence of allergic mechanisms. Angiotensin Ⅱ receptor blocker (ARB), a relatively new class of anti-hypertensive drug, has rarely been reported to induce severe angioedema [7]. Upper airway edema due to angioedema is a potentially life-threatening condition requiring prompt recognition and management [8]. The rapid accumulation of fluid in the submucosal tissue leads to significant swelling of the upper airway structures, including the tongue, pharynx, and larynx. This can cause partial or complete airway obstruction, necessitating immediate intervention to prevent respiratory failure. A previous study reported that 0.1-0.7% of patients on ACE inhibitors develop angioedema, with up to 22-35% of those cases potentially developing significant airway obstruction [8]. In cases where drug-induced causes are suspected, discontinuation of medications is critical. In the present patient, his recent history of excision of ranula and intake of ARB were considered to be triggers of angioedema; therefore, we immediately discontinued ARB after recognition of his condition.

Management of upper airway edema with respiratory distress due to angioedema involves securing the airway, which often requires advanced techniques, such as video laryngoscopy or fiberoptic intubation, or surgical intervention, such as tracheotomy. Securing the airway at the emergency department presents unique challenges that demand prompt and effective intervention. The present case highlights several key considerations and interventions relevant to emergency airway management. A previous study reported that the incidence of difficult airway management outside the operating room ranged from 4.2% to 10.3% [1]. This enforces the need for specialized training and equipment to address such cases effectively.

**Figure 3:**
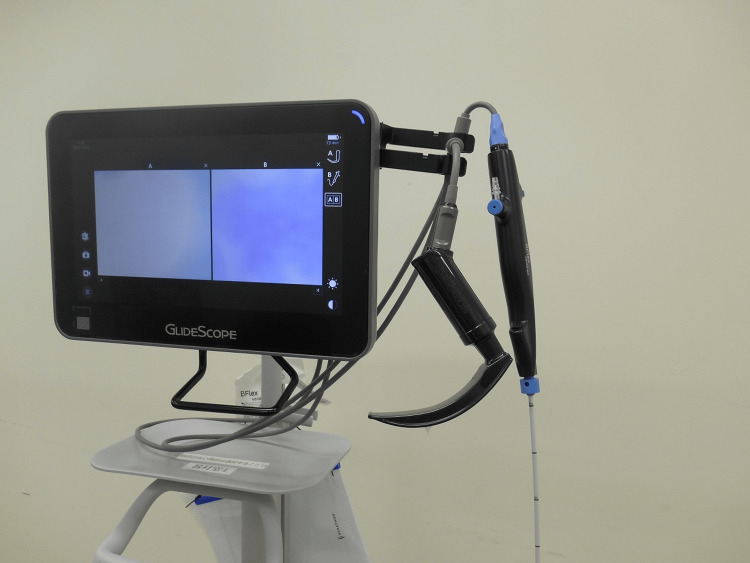
GlideScope Core™ equipped with dual-video laryngoscope and bronchoscope

**Figure 4:**
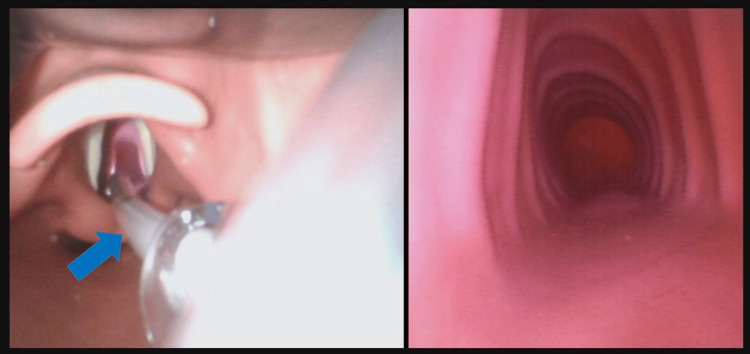
Dual view of the video laryngoscope and bronchoscope displayed on the GlideScope Core™ system Display of the video laryngoscope showing the bronchoscope (arrow) passing through the glottis (left) and the bronchoscope view visualizing the inside of the trachea (right). These images were captured using an airway simulator.

The choice of airway devices is crucial in emergency airway management. Although the usefulness of video laryngoscopes compared to direct laryngoscopes in the emergency department has been reported [9], the latest combined video laryngoscope and bronchoscope system, GlideScope Core™ (Figures 3-4), offers excellent visualization of the airway by simultaneous use of a video laryngoscope and bronchoscope in a dual-view. Formerly, video laryngoscope and bronchoscope were used separately for video-laryngoscope-assisted bronchoscopic intubation in anticipated difficult airway patients [10,11]. This portable device combines the benefits of a video laryngoscope and a bronchoscope in a single platform, offering flexibility in airway management strategies in the operating room and at the bedside, including in the emergency department. The hybrid functionality is particularly useful in complex cases where visualization and maneuverability are essential. Furthermore, rapidly switching between the two devices allows the operators to adapt their approach in real time, enhancing successful rates for difficult intubations. In the present case, despite encountering difficulty with initial intubation attempts using a video laryngoscope, the availability of an alternative device, a bronchoscope, allowed for successful airway management. This emphasizes the importance of maintaining a diverse armamentarium of airway devices and techniques for effectively managing challenging airway scenarios [12]. Although advanced airway devices are invaluable for managing difficult airway cases, surgical airway management should always be considered.

## Conclusions

We successfully secured the airway of a patient with severe upper airway edema due to angioedema. Although the use of a video laryngoscope has become standard practice even for routine endotracheal intubation, this hybrid device, which combines the functions of a video laryngoscope and a bronchoscope, is expected to become increasingly prevalent as a solution for managing difficult airways to prevent fatal outcomes.
